# Serial expression analysis of breast tumors during neoadjuvant chemotherapy reveals changes in cell cycle and immune pathways associated with recurrence and response

**DOI:** 10.1186/s13058-015-0582-3

**Published:** 2015-05-29

**Authors:** Mark Jesus M. Magbanua, Denise M. Wolf, Christina Yau, Sarah E. Davis, Julia Crothers, Alfred Au, Christopher M. Haqq, Chad Livasy, Hope S. Rugo, I-SPY 1 TRIAL Investigators, Laura Esserman, John W. Park, Laura J. van ’t Veer

**Affiliations:** Helen Diller Family Comprehensive Cancer Center, University of California San Francisco, San Francisco, CA USA; Division of Hematology/Oncology, University of California San Francisco, Box 1387, 2340 Sutter Street, San Francisco, CA 94115 USA; Department of Laboratory Medicine, University of California San Francisco, San Francisco, CA USA; Department of Surgery, University of California San Francisco, San Francisco, CA USA; Buck Institute for Research on Aging, Novato, CA USA; Department of Urology, University of California San Francisco, San Francisco, CA USA; UNC Lineberger Comprehensive Cancer Center, University of North Carolina, Chapel Hill, NC USA

## Abstract

**Introduction:**

The molecular biology involving neoadjuvant chemotherapy (NAC) response is poorly understood. To elucidate the impact of NAC on the breast cancer transcriptome and its association with clinical outcome, we analyzed gene expression data derived from serial tumor samples of patients with breast cancer who received NAC in the I-SPY 1 TRIAL.

**Methods:**

Expression data were collected before treatment (T1), 24–96 hours after initiation of chemotherapy (T2) and at surgery (TS). Expression levels between T1 and T2 (T1 vs. T2; n = 36) and between T1 and TS (T1 vs. TS; n = 39) were compared. Subtype was assigned using the PAM50 gene signature. Differences in early gene expression changes (T2 − T1) between responders and nonresponders, as defined by residual cancer burden, were evaluated. Cox proportional hazards modeling was used to identify genes in residual tumors associated with recurrence-free survival (RFS). Pathway analysis was performed with Ingenuity software.

**Results:**

When we compared expression profiles at T1 vs. T2 and at T1 vs. TS, we detected significantly altered expression of 150 and 59 transcripts, respectively. We observed notable downregulation of proliferation and immune-related genes at T2. Lower concordance in subtype assignment was observed between T1 and TS (62 %) than between T1 and T2 (75 %). Analysis of early gene expression changes (T2 − T1) revealed that decreased expression of cell cycle inhibitors was associated with poor response. Increased interferon signaling (TS − T1) and high expression of cell proliferation genes in residual tumors (TS) were associated with reduced RFS.

**Conclusions:**

Serial gene expression analysis revealed candidate immune and proliferation pathways associated with response and recurrence. Larger studies incorporating the approach described here are warranted to identify predictive and prognostic biomarkers in the NAC setting for specific targeted therapies.

**Clinical trial registration:**

ClinicalTrials.gov identifier: NCT00033397. Registered 9 Apr 2002.

**Electronic supplementary material:**

The online version of this article (doi:10.1186/s13058-015-0582-3) contains supplementary material, which is available to authorized users.

## Introduction

Women with locally advanced or high-risk early-stage breast cancers are eligible for neoadjuvant chemotherapy (NAC), an approach whereby patients receive systemic chemotherapy before surgical removal of the tumor. NAC can downstage tumors to allow breast-conserving surgery to be performed [1, 2] and permits the evaluation of individual tumor response to monitor the effectiveness of standard and/or investigational systemic therapy [3–6]. Recent clinical studies involving NAC have provided an opportunity to evaluate prognosis based on the presence of residual disease and to elucidate predictors of response to different types of chemotherapeutics [7–11]. These studies have consistently shown that cancers in women who respond to NAC with a pathological complete response (pCR) are much less likely to recur than those in women with residual disease [5]. Unfortunately, only a subset of patients achieve a pCR to neoadjuvant treatment [12–14]. The results of a large meta-analysis were recently reported [15]. To date, there are no genomic markers that can predict response to NAC [16, 17].

Why many patients fail to respond to NAC is poorly understood. Questions remain about which genes and pathways in breast tumors are perturbed in response to treatment [8, 11] and how these molecular signals differ between responders and nonresponders, as well as in those whose cancer recurs early vs. those whose cancer does not. For example, early changes in the proliferation marker Ki-67 have been found to correlate positively with pathological response [18]. Compared with pretreatment levels [19], post-NAC Ki-67 levels appeared to show a stronger relationship with recurrence-free survival (RFS) [20]. In addition to single-gene studies, genomic approaches are needed to maximize the discovery of useful classifiers and druggable targets in the NAC setting.

We performed an exploratory study to investigate the dynamics of tumor gene expression in early high-risk breast cancer patients receiving NAC. We analyzed serial cDNA microarray expression data obtained from breast tumor biopsies before treatment (T1), at 24–96 hours after the first dose of NAC (T2) and in residual tumors at the time of surgery (TS). We assessed differentially expressed genes between time points (T1 vs. T2 and T1 vs. TS) as well as changes in pathways and molecular subtypes. We also explored associations between early gene expression changes (T2 − T1) and response to chemotherapy, as well as gene expression in residual tumors (TS and TS − T1) and recurrence.

## Methods

### Ethics, consent and permissions

The protocol for the Investigation of Serial Studies to Predict Your Therapeutic Response with Imaging And moLecular Analysis (I-SPY 1 TRIAL; registration numbers CALGB 150007/150012, ACRIN 6657) was approved by the institutional review boards at all participating institutions [7] (Additional file 1). All patients signed informed consent forms to allow molecular analyses to be performed on their tissue samples.

### Patients and tissue samples

**Fig. 1:**
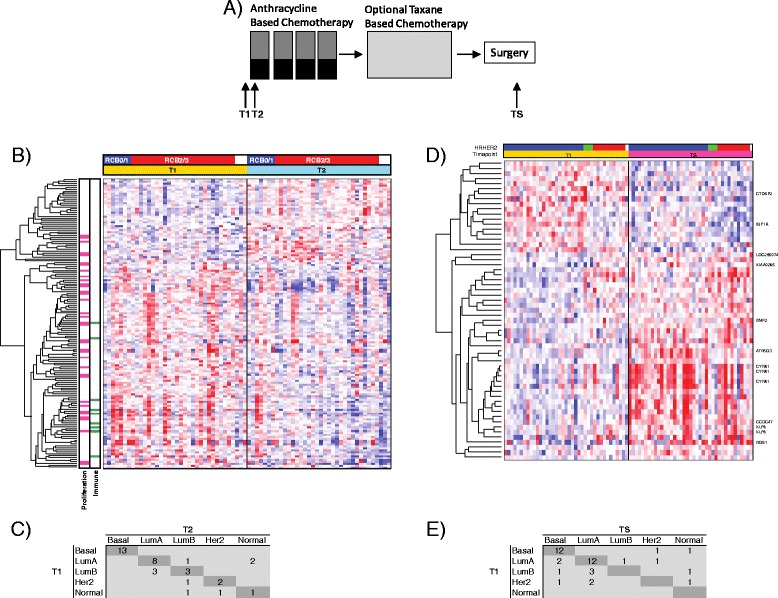
Serial gene expression analysis in locally advanced breast cancer patients undergoing neoadjuvant chemotherapy (NAC). **a** Study schema. Gene expression analysis was performed on breast cancer tumors collected before treatment (*T1*), 24–96 hours after initiation of anthracycline-based NAC (*T2*) and at the time of surgery (*TS*). **b** Heat map showing results of supervised clustering analysis of expression profiles of tumors before treatment (*T1*) and 24–96 hours after initiation of NAC (*T2*). *Rows* indicate expression levels for each gene, and *columns* indicate individual samples. *Blue* indicates downregulation of gene expression, and *red* indicates upregulation of gene expression. The *upper color bar* indicates response to NAC as defined by residual cancer burden (RCB 0/I or RCB II/III). *Bars* on the *left* indicate assignment of genes to an ontology group (i.e., immune system– or proliferation-related genes). *Her2* Human epidermal growth factor receptor 2, *LumA* luminal A, *Lum B* luminal B. **c** Subtype assignments of matched tumors at T1 and T2. *Dark gray boxes* running diagonally downward from *top left* indicate no change between two time points. **d** Heat map showing result of supervised clustering analysis of expression profiles of known nonresponding tumors at T1 and TS. The *upper color bar* indicates hormone receptor (HR) and HER2 status (*blue* = HR^+^HER2^−^; *green* = HR^−^HER2^+^; *red* = HR^−^HER2^−^; *white* = no data). The 10 most significant differentially expressed genes are indicated at the *right* of the heat map. *Blue* indicates downregulation of gene expression, and *red* indicates upregulation of gene expression. **e** Subtype assignments of matched tumors at T1 and TS. *Dark gray* boxes running diagonally downward from *top left* indicate no change between two time points

Eligible patients were women with histologically confirmed invasive breast tumors greater than 3 cm in diameter with no evidence of distant metastatic disease (Table 1). Figure 1a shows the study schema. Core needle (16-gauge) biopsies were taken from the primary breast tumors before treatment (T1) and between 24 and 96 hours after the first dose (T2) of chemotherapy. After four cycles of anthracycline-based therapy, patients received a taxane regimen followed by surgery, when another biopsy was taken (TS). Collected tissue samples were immediately frozen in Tissue-Tek O.C.T*.*™ embedding media (Sakura Finetek, Alphen aan den Rijn, the Netherlands) and then stored at −80 °C until further processing. Using a cryostat, four 14-μm sections were obtained for RNA isolation. An additional 8-μm section was stained with hematoxylin and eosin (H&E), and a histopathological evaluation was performed (by AA) to demarcate tissue containing at least 50 % tumor. The H&E-stained slide was then used as a guide to macrodissect tumors on the remaining nonstained dehydrated sections.

**Table 1:** Patient and clinical characteristics

Characteristics	I-SPY 1 TRIAL evaluable patients (n = 221)	Matched T1-T2 pairs (n = 36)	*P*-value	Matched T1-TS pairs (n = 39)	*P*-value
Age, yr			0.77^a^		0.86^a^
Median (range)	49 (26–28)	47 (31–68)		48 (34–65)	
Clinical tumor size, cm					
Median (range)	6 (0–25)^c^	6 (3–17)	0.55^a^	6 (0–25)^c^	0.88^a^
Clinically node-positive at diagnosis	143 (65 %)	25 (69 %)		26 (67 %)	
Histological grade (baseline)					
Low	18 (8 %)	1 (3 %)	0.39^b^	4 (10 %)	0.83^b^
Intermediate	96 (43 %)	20 (56 %)		17 (44 %)	
High	103 (47 %)	15 (42 %)		18 (46 %)	
Indeterminate	4 (2 %)	0		0	
Clinical stage (baseline)					
I	3 (1 %)	0	0.80^b^	0	0.66^b^
II	104 (47 %)	15 (42 %)			
III	96 (43 %)	18 (50 %)		16 (41 %)	
Inflammatory	17 (8 %)	3 (8 %)		5 (13 %)	
Indeterminate	1 (<1 %)	0		0	
Hormone receptor-positive (ER or PR) at baseline	131 (59 %)	24 (67 %)	0.55^b^	26 (68 %)	0.41^b^
HER2-positive at baseline	67 (31 %)	6 (17 %)	0.12^b^	3 (8 %)	0.006^b^
Neoadjuvant treatment					
AC only	11 (5 %)	3 8 %)	0.21^b^	2 (5 %)	0.38^b^
AC + taxane	187 (85 %)	33 (92 %)		33 (85 %)	
AC + T + Trastuzumab	20 (9 %)	0		2 (5 %)	
AC + T + Other	3 (1 %)	0		2 (5 %)	
Surgery type					
Mastectomy	123 (56 %)	19 (53 %)	0.95^b^	22 (56 %)	0.58^b^
Lumpectomy	92 (41 %)	16 (44 %)		17 (44 %)	
No surgery	6 (3 %)	1 (3 %)			
RCB					
0	56 (25 %)	6 (17 %)	0.29^b^	0	0.0002^b^
I	18 (8 %)	1 (3 %)		1 (3 %)	
II	86 (39 %)	16 (44 %)		18 (46 %)	
III	41 (19 %)	10 (28 %)		16 (41 %)	
Undetermined	20 (9 %)	3 (8 %)		4 (10 %)	

^a^Wilcoxon rank-sum test. ^b^χ^2^ test. ^c^One patient had a tumor that measured 0 cm with calipers by clinical examination but had a >3-cm tumor revealed by magnetic resonance imaging (MRI) and was therefore eligible for the trial. *Abbreviations*: *AC* anthracycline, *ER* estrogen receptor, *HER2* Human epidermal growth factor receptor 2, *PR* progesterone receptor, *RCB* residual cancer burden

### cDNA microarray gene expression profiling

Total RNA isolation, amplification and cDNA microarray analysis have been previously described [21, 22]. The microarray was composed of 39,347 cDNAs representing 20,862 annotated genes. Clone annotation was updated using the SOURCE database [23], and the UCSC Genome Browser database and software tools [24] were used for clones without UniGene annotations. The quality of the arrays was analyzed using the arrayQuality package in Bioconductor.

Amplified RNA samples were hybridized to six different batches of printed cDNA microarrays. Arrays in each print batch were normalized, and data from different batches were combined by mapping using gene symbols and averaging probe values from replicate genes. The print run effect was then controlled for by fitting a linear model with the response expressed as the log_2_ ratio and batch effect as the explanatory variable and using the residuals from the fit for further analyses [21]. The microarray data were deposited in the Gene Expression Omnibus database [GEO:GSE32603].

### Molecular subtype assignment

Each sample was assigned to a molecular subtype using the PAM50 method [25]. Concordance of molecular subtype assignment of paired samples between time points was calculated by dividing the number of matched pairs with identical assignment by the total number of matched pairs.

### Identifying changes in expression between different time points

To determine genes that were differentially expressed between time points (T1 vs. T2 and T1 vs. TS), the resulting normalized dataset was analyzed using a paired sample permutation test based on the *t*-statistic. The Benjamini-Hochberg false discovery rate adjustment (multitest package in R) was applied to correct for multiple comparisons. Probes with adjusted *P*-values less than 0.05 were considered significant.

### Association of changes in gene expression with clinical endpoints

Outcome parameters included residual cancer burden (RCB) after therapy and RFS. RCB categories assigned at the surgery time point were retrospectively used to compare gene expression between T1 and T2. Owing to a limited sample size, we divided patients into two groups: RCB 0/I and RCB II/III. RCB 0/I was defined as no or minimal residual tumor cells in both the breast and axillary lymph nodes, and RCB II/III was used to denote nonresponse with moderate to extensive residual disease [6]. At T2, tumor tissue was still available for analysis in patients eventually classified as RCB 0/I. Differential expression analysis between RCB 0/I and RCB II/III at T1, T2 and T2 − T1 was performed using a permutation test based on the *t*-statistic. Cox proportional hazards modeling was used to identify genes associated with RFS. For analyses involving TS and TS − T1, we included only data from nonresponding patients (i.e., RCB I/II/III). Owing to the small sample sizes, we adopted a relaxed significance threshold for outcome associations (*P* < 0.005 without correction for multiple testing). To illustrate the association with RFS using Kaplan-Meier survival analysis, genes that were significantly associated with RFS as continuous variables were dichotomized as top vs. combined middle and bottom tertiles.

### Assessment of Ki-67 expression

Immunohistochemical (IHC) staining to assess Ki-67 protein expression was performed as previously described using a standard avidin-biotin complex technique [5]. IHC staining was done centrally, and a single pathologist (CL) interpreted the results. The percentage of Ki-67-positive nuclei was quantified using the Aperio Nuclear V9 (cell quantification) algorithm (Leica Biosystems, Buffalo Grove, IL, USA). Each case was assigned a Ki-67 score, defined as the percentage of total number of tumor cells positive for nuclear staining by the antibody.

### Pathway analysis

Ingenuity Pathway Analysis (IPA; Ingenuity Systems/Qiagen, Redwood City, CA, USA) was used to map lists of significant genes to gene ontology groups and biological pathways. IPA then uses Fisher’s exact test to calculate a probability value to indicate the association between each gene in the list and IPA-curated pathways and biological functions. A *P*-value less than 0.05 was considered statistically significant overrepresentation of genes in a canonical pathway or gene ontology group (e.g., molecular and cellular functions).

## Results

### Patient and microarray data

Patient characteristics are summarized in Table 1. Of the 237 patients enrolled, 221 patients completed NAC, of whom 56 (25 %) achieved a complete pathological response (Additional file 2). Arrays were available for 141, 45 and 54 patients corresponding to time points T1, T2 and TS (Fig. 1a). Paired expression data for T1 vs. T2 and T1 vs. TS were available for 36 and 39 patients, respectively. The patients in this study had clinical characteristics similar to those of the whole patient cohort in the I-SPY 1 TRIAL [7], except for fewer human epidermal growth factor receptor 2 (HER2)-positive tumors. Also, a different distribution of RCB cases was observed because only nonresponders were included for TS.

### Differentially expressed genes, pathways and molecular subtype before treatment vs. 24–96 hours after initiation of chemotherapy

To determine perturbations in gene expression after the first dose of chemotherapy, we performed differential expression analysis between pretreatment (T1) and post–cycle 1 (T2) biopsies. The results revealed significantly altered expression of 150 probes (124 genes) (Fig. 1b and Additional file 3). Interestingly, most genes were downregulated at T2 (106 probes, 93 genes). Gene ontology enrichment analysis revealed a significant overrepresentation of genes involved in cell cycle and in cell death and survival (Additional file 4). These included genes encoding kinases, such as *AURKA* and *PLK1*, which play key roles in cell proliferation. Noteworthy also was downregulation of immune function–related genes such as *HLA-DOA*, *HLA-DQA1*, *TLR7*, *TLR8* and *MAP3K14*. In pathway analysis, we identified 35 enriched canonical pathways, including immune signaling, p53 signaling, mammalian target of rapamycin signaling, cell cycle signaling and carbohydrate metabolic pathways. Of note, most of the modulated pathways were immune-related (e.g., altered T cell and B cell signaling in rheumatoid arthritis, dendritic cell maturation and Toll-like receptor and TREM1 signaling), suggesting the suppression of immune function at this early time point.

Comparison of molecular subtypes of paired samples between time points T1 and T2 revealed changes in 9 of the 36 assignments (concordance rate = 75 %) (Fig. 1c). Interestingly, half of the luminal B subtypes converted to luminal A, whereas all 13 basal tumors remained unchanged.

### Early gene expression changes associated with response to chemotherapy

We used a permutation test based on the *t*-statistic to identify associations between early gene expression changes (T2 − T1) and responses (RCB 0/I vs. RCB II/III). RCB scores were available for 33 of the 36 patients with paired T1 and T2 arrays. Because the sample sizes were small (7 RCB 0/I and 26 RCB II/III cases), we adopted a relaxed significance threshold (permutation *P* < 0.005 without correction for multiple testing). The results revealed 123 probes (97 genes) with significantly different early gene expression changes (T2 − T1) between the two RCB groups (Additional file 5). A total of 49 of these probes (30 genes) showed higher expression changes within the RCB II/III cases, whereas 74 probes (67 genes) had higher expression changes within the RCB 0/I class. We observed a significant overrepresentation of cell cycle genes associated with poor response (Additional file 6).

**Fig. 2:**
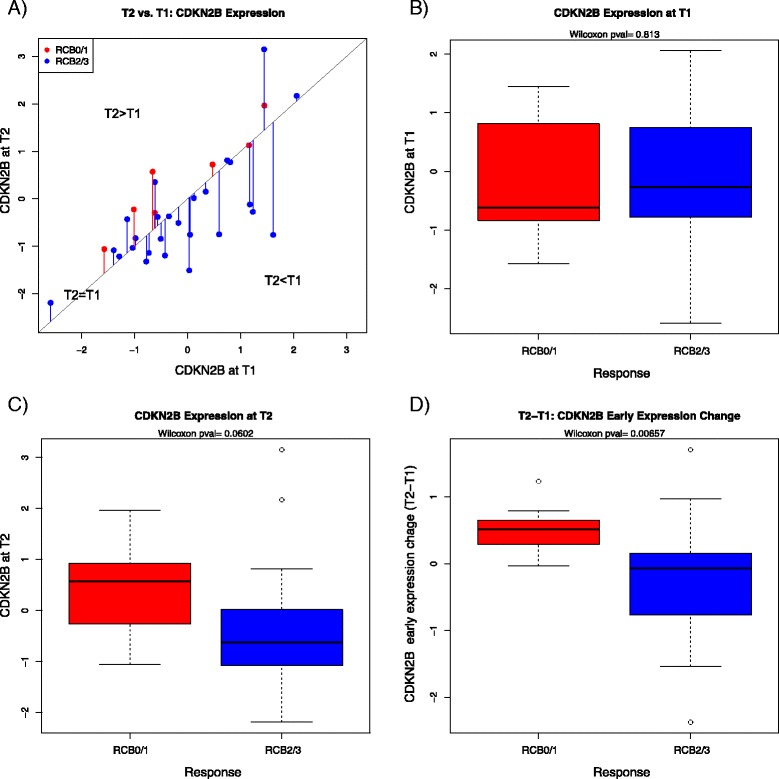
Association between gene expression and response to chemotherapy as defined by residual cancer burden (RCB). **a** Scatterplot showing the expression of a representative cell proliferation gene, *CDKN2B*, at baseline (T1) and 24–96 hours after initiation of chemotherapy (T2) with the color of the points indicating response [*red* = RCB 0/I (responders); *blue* = RCB II/III (nonresponders)]. The *diagonal line* represents no change in gene expression levels (T1 = T2). The length of the *vertical lines* between points and the diagonal line represents the magnitude of expression change from T1 to T2. **b**–**d** Box plots show expression of *CDKN2B* in RCB 0/I and RCB II/III at T1 (**b**), at T2 (**c**) and the change in gene expression between two time points (T2 − T1) (**d**)

In pathway analysis, we identified 20 canonical pathways significantly associated with early changes in gene expression, including several pathways involved in amino acid metabolism (e.g., glycine biosynthesis and lysine degradation pathways). Interestingly, the translation repressor *EIF4EBP1* and the cell cycle inhibitors *CDKN2B* and *SMARCB1* showed decreased expression at T2 relative to T1 within the RCB II/III subset, suggesting an association between decreased gene expression of negative regulators of the cell cycle and poor response to chemotherapy. Figure 2 shows an example where dynamic change [i.e., decreased expression of *CDKN2B* between two time points (T2 − T1)] was significantly associated with poor response, whereas the pretreatment (T1) and post–cycle 1 (T2) levels did not.

Next, we examined whether genes associated with chemotherapy response at static time points (T1 and T2) were similar to those observed when change in expression between two time points was considered (T2 − T1). The results revealed fewer genes associated with response at T1 (17 probes, 12 genes) or T2 (115 probes, 87 genes) as compared with change in expression (T2 − T1: 123 probes, 97 genes) (Additional file 5). In addition, the overlap between the gene sets was minimal or nonexistent (Additional file 2), suggesting that expression profiling of tumors before treatment and early during therapy, as well as the assessment of early changes in expression, may provide nonredundant information regarding chemotherapy responsiveness.

### Pretreatment versus residual tumor gene expression in nonresponders

We performed differential expression analysis between the pretreatment biopsy (T1) and the surgical specimen (TS) to identify genes with significantly altered expression in residual tumors. The results revealed the significant modulation of 59 probes (47 genes) (Fig. 1d and Additional file 7). Gene ontology enrichment analysis revealed the significant overrepresentation of genes involved in cell movement, cell death and survival (Additional file 8). Of note, *CYR61*/*IGFBP10*, an extracellular matrix–associated signaling protein and components of the key transcriptional regulator AP-1, *JUN* and *FOSB* were among the 32 upregulated genes in residual tumors after chemotherapy. Pathway analysis revealed 54 canonical pathways that were significantly modulated at TS, including regulation of the epithelial–mesenchymal transition pathway, insulin-like growth factor 1 (IGF1) signaling and immune-related signaling, such as interleukin (IL)-8 and IL-1 signaling. Comparison of molecular subtypes of paired samples between time points T1 and TS revealed changes in 15 of the 39 assignments (concordance rate = 62 %) (Fig. 1e). Two of the fourteen basal subtypes changed to HER2 and normal-like.

### Late gene expression changes associated with recurrence in nonresponders

**Fig. 3:**
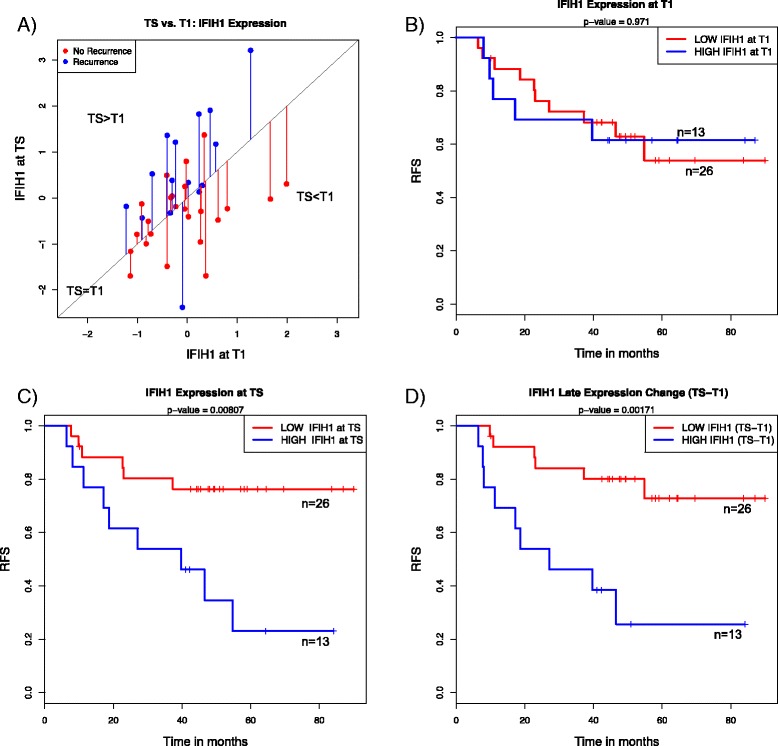
Association of changes in gene expression between pretreatment and residual tumors (TS − T1) and recurrence-free survival (RFS). **a** Scatterplot showing the expression of a representative interferon signaling gene, *IFIH1*, at TS and T1, with the color of the points indicating outcome (*red* = no recurrence; *blue* = recurrence). The *diagonal line* represents no change in gene expression levels (T1 = TS). The length of the *vertical lines* between points and the *diagonal line* represents the magnitude of expression change from T1 to TS. Kaplan-Meier analysis of RFS among patients with high (top tertile: *blue*) or low expression (*red*) levels of *IFIH1* at T1 (**b**), at TS (**c**) and the change in gene expression between two time points (TS − T1) (**d**)

We examined associations between RFS and expression changes between pretreatment and time of surgery (TS − T1) using Cox proportional hazards modeling. We identified late expression changes in 95 probes (57 genes) associated with RFS (Additional file 9). These were enriched for increased expression of interferon signaling genes, such as *IFIT2*, *IFIT1*, *IFITM1*, *IFIH1* and *EML2*. This observation is consistent with results of the pathway analysis showing that interferon signaling was the most enriched canonical pathway in the overall set (Additional file 10). To illustrate the associations with RFS, we dichotomized patients based on expression levels at T1, TS and TS − T1. Figure 3 shows examples where increased expression of *IFIH1* over the course of NAC (TS − T1) was significantly associated with early recurrence. Interestingly, similar associations with RFS were observed for elevated levels of *IFIH1* in surgical specimens (TS), but not for pretreatment (T1) levels.

### Residual tumor expression at time of surgery associated with recurrence in nonresponders

**Fig. 4:**
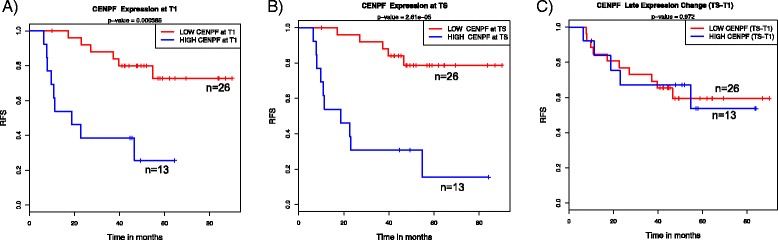
Association between gene expression in residual tumors (TS) and recurrence-free survival (RFS). Kaplan-Meier analysis of RFS among patients with high (top tertile: *blue*) or low expression (*red*) levels of a representative cell proliferation gene, *CENPF*, at T1 (**a**), at TS (**b**) and the change in gene expression between two time points (TS − T1) (**c**)

We examined the associations between gene expression as continuous variables in residual tumors at TS and RFS using Cox proportional hazards modeling. Expression of 181 probes (127 genes) was found to be associated with RFS. Of these, high expression of 129 probes (93 genes) and low expression of 52 probes (34 genes) were associated with reduced RFS (Additional file 9). Gene ontology analysis revealed significant enrichment of genes related to the cell cycle (Additional file 11). For instance, high expression levels of the cell proliferation genes *CENPF*, *AGR2* and *E2F3* in the patients with residual disease were associated with poor outcomes. Of note, overexpression of *TGFBI* and interferon-induced proteins *IFIT2*, *IFIH1* and *IFI44L* in the residual tumor were also associated with reduced RFS. Pathway analysis revealed significant enrichment for immune-related functions (e.g., IL-2 and IL-6 signaling). Figure 4 shows examples where dichotomized expression levels of *CENPF* in surgical specimens (TS) were associated with recurrence. Interestingly, high pretreatment (T1) levels of *CENPF* were also associated with recurrence, whereas late changes in expression (TS − T1) were not.

Next, we examined whether genes that were associated with RFS at pretreatment (T1) were similar to those observed at TS or when late changes in gene expression (TS − T1) were considered. We identified more T1 genes (260 probes, 183 genes) associated with recurrence compared with those observed for TS − T1 (95 probes, 57 genes) or TS (181 probes, 127 genes) (Additional file 9). Also, only a few genes were found to be in common when any two gene sets were compared, and no overlap was observed when all three gene sets were considered (Additional file 2). These results suggest that expression profiling at static time points (e.g., at pretreatment only and at surgery only) and assessment of expression changes between two time points may provide nonredundant information regarding the likelihood of recurrence in nonresponders.

### Ki-67 protein expression

**Fig. 5:**
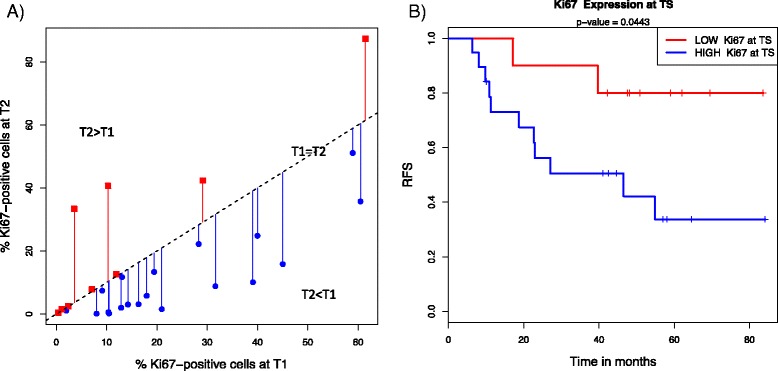
Assessment of Ki-67 protein expression. **a** Scatterplot showing Ki-67 scores at baseline (T1) and 24–96 hours after initiation of chemotherapy (T2), with the colors and shapes of the points indicating increases (*red squares*) or decreases (*blue circles*). The *diagonal line* represents no change in Ki-67 score (T1 = T2). The length of the *vertical lines* between points and the *diagonal line* represents the magnitude of expression change from T1 to T2. **b** Kaplan-Meier analysis of recurrence-free survival (RFS) among patients with low (bottom tertile: *red*) or high (*blue*) Ki-67 scores at TS

Of the 36 patients with paired T1 and T2 expression data, 28 had matched Ki-67 scores. Consistent with the observed downregulation of proliferation signals at T2, the percentage of Ki-67-positive cells decreased from T1 to T2 (*P* = 0.020 by Wilcoxon paired test) (Fig. 5a). When changes in paired Ki-67 scores between the two time points (T2 − T1) were considered, no significant association with chemotherapy response was observed (*P* = 0.66). Paired Ki-67 scores were available for 29 of the 39 patients with matched T1 and TS expression data. Cox proportional hazards modeling revealed that higher Ki-67 scores at TS [*P* = 0.0061 by likelihood ratio (LR) test] or at T1 (*P* = 0.031 by LR test) were associated with decreased RFS. Figure 5b shows the results of the Kaplan-Meier analysis of RFS among patients with low (bottom tertile: red) or high (blue) Ki-67 scores at TS. Changes in Ki-67 scores between T1 and TS showed a trend toward association with RFS, but did not reach significance (*P* = 0.075 by LR test).

## Discussion

The impact of NAC on the biology of breast cancers is not well understood. Recent clinical studies have included correlative gene expression analyses to understand the effects of NAC on the breast tumor transcriptome [8, 11, 26–29]. For example, Hannemann and colleagues [11] compared gene expression before and after NAC and observed that sensitive tumors showed significant changes in their gene expression, whereas resistant tumors did not. Gonzalez-Angulo and colleagues [8] found that different pathways were preferentially perturbed in basal-like vs. non-basal-like breast cancers. In prior gene expression profiling studies, researchers have also attempted to identify candidate predictive markers for chemotherapeutic response using pretreatment biopsies [30–32]. However, this approach does not account for chemotherapy-induced perturbations that may be gleaned from serial gene expression analysis of tumors in patients undergoing neoadjuvant treatment. Most importantly, examination of serial changes in tumor gene expression may identify predictors for favorable outcome with better sensitivity than baseline signatures alone [33, 34].

In the present study, we examined changes in gene expression in serial breast tumor biopsies and the surgical specimen treated with NAC, as well as their association with clinical outcomes. We performed serial cDNA microarray profiling before, during and after treatment. We compared gene expression and molecular subtype assignment of matched tumors collected at different time points. We also performed exploratory analyses to evaluate associations between early changes in gene expression and response as well as expression in residual tumors and recurrence. To our knowledge, this is the first study of changes in tumor gene expression over three different time points in the neoadjuvant setting.

Transcriptomic analysis in breast cancer tumors revealed genes and pathways that were significantly perturbed after initiation of first cycle of NAC. Early effects of NAC on tumor gene expression include a substantial downregulation of genes and pathways involved in proliferation and immune function. These results must be interpreted with caution, however, as tissue biopsies were collected over a 3-day period. Therefore, confounding factors, including treatment-associated changes in the stroma and immune cell infiltrates, may potentially influence gene expression at this time point (T2).

The expression of the proliferation marker Ki-67 was evaluated by immunohistochemistry in a small subset of paired samples. Ki-67 expression decreased early during therapy, which is consistent with the observed downregulation of proliferation genes as determined by microarray analysis.

Residual tumors after NAC also showed significant modulation of genes and pathways involved in immune response pathways. These observations are consistent with the known immune-suppressive and cytotoxic effects of chemotherapy and the steroids that are often coadministered [2, 35]. Genes and pathways significantly altered in residual tumors may represent candidate markers for chemoresistance [36]. For example, perturbations in the expression of genes involved in IGF-1 signaling (e.g., the upregulation of *CYR61*) have been observed in previous studies in which gene expression in pre- and post-NAC biopsies was compared [11, 36]. Interestingly, the gene *CYR61* (*IGFBP10*) has been shown to be associated with breast cancer progression [37, 38] and with resistance to apoptosis [39] and can be a potential target for therapy [40].

We observed changes in molecular subtypes of tumors in a subset of patients during the course of NAC. Discordance in subtype assignment was higher when pretreatment tumors were compared with matched residual tumors at the time of surgery (T1 vs. TS) than with tumors obtained early in treatment (T1 vs. T2). The most frequent change in molecular subtype that we observed after cycle 1 of NAC was from luminal B to luminal A. Korde and colleagues reported similar findings [31]. The switch from luminal B to luminal A may reflect the selective killing of highly proliferative cells that are chemotherapy-sensitive, leaving behind tumor cells that are more hormone-sensitive and less responsive to chemotherapy [31]. Gonzales-Angulo et al. [8] reported a similar concordance rate of subtypes (62 %) of paired pre- and post-NAC samples (n = 21) (Additional file 12). The lower concordance for T1 vs. TS than for T1 vs. T2 may reflect the much larger effect of the full regimen of NAC on the cellularity and composition of tumor tissue. It is unclear, however, whether the interconversion between non-basal-like subtypes is a result of chemotherapy exposure or is due to the relative instability in these subtypes as compared with the basal-like subtypes [8]. Finally, our results regarding changes in molecular subtype assignment must be interpreted with caution, owing to the small sample size and the lack of consensus for molecular subtype assignment. Furthermore, changes in subtype can be attributed to reduced proliferation and changes in cellularity in both the tumor and the stroma. It is currently impossible to distinguish among these possibilities, and, even so, the clinical utility of the observation remains unclear.

Exploratory analysis comparing expression profiles of responders vs. nonresponders revealed differentially expressed genes involved in amino acid metabolism and cell proliferation. The relationship between amino acid metabolism and response to chemotherapy is currently unclear. The results of a recent preclinical study suggested, however, that the activation of amino acid metabolic pathways might be important in acquiring resistance to chemotherapy [41]. We have shown in our previous work that high Ki-67 expression (n = 166 patients) at T1 was associated with favorable response to chemotherapy [5]. In this study involving a small subset of patients with paired expression data, we did not observe a significant relationship between Ki-67 scores and response (*P* = 0.18). Interestingly, we found that, early during chemotherapy, decreased expression of cell cycle inhibitors rather than increased expression of positive regulators of cell cycle (e.g., Ki-67 [18]) was associated with poor response.

Molecular analysis of residual tumors may provide prognostic and predictive information and may facilitate the development of biomarkers, along with efficacious single-agent and combination therapies, to prevent or delay recurrence. Currently, there are no genomic predictors to determine which patients will experience an early recurrence [7]. We found that increased interferon signaling over the course of chemotherapy among nonresponding patients was associated with shorter RFS, and we speculate that it may represent an immune tolerance response in aggressive disease. This seemingly contradictory relationship between interferon signaling and poor outcome vs. the more typically reported associations between T cell–B cell immune system signals and good outcome has been well documented [42]. For example, a recent study has shown that activated interferon signaling is associated with increased risk of distant metastasis among luminal subtype tumors [43]. Interferon signaling has also been associated with resistance to chemotherapy and radiation treatment [44], and it has been identified as a coexpression module independent of other immune signaling in breast and other types of cancers [45, 46]. Taken together, our results suggest that treatment-induced changes in interferon pathway signaling may be an important component in assessing risk factors for breast cancer recurrence and may hint at the potential utility of immune modulating therapy in nonresponding patients.

Examining changes in expression (e.g., T2 − T1 and TS − T1) may provide nonredundant information regarding response and recurrence beyond those obtained from static time points alone. Integrating gene expression data from different time points, including the changes observed between them, may facilitate the development of improved predictors for poor response and early recurrence.

Molecular markers of response and survival can vary across breast cancer subtypes. Therefore, molecular subtypes need to be considered when evaluating associations between tumor expression and clinical outcomes. In this study, the sample size was too small to perform subset analysis, but we found similar results in models adjusted for hormone receptor (HR) status. For example, analyses with or without adjustment for HR status revealed a significant association between RFS and interferon signaling genes (e.g., *IFIT2*, *IFIH1* and *IFI44L*). Likewise, the gene expression changes that were most highly associated with response (e.g., *CDKN2B*, *EIF4EBP1*) were similar in both univariate and HR adjusted models.

A limitation of the present study was the relatively small sample size. Therefore, we consider our analysis to be exploratory, and larger studies are warranted to validate our findings. A similar study incorporating the approaches described here will be performed within the I-SPY 2 TRIAL [47]. This neoadjuvant clinical trial is particularly well designed to test and develop predictive biomarkers because participating patients undergo serial tumor biopsies before treatment, after three cycles of neoadjuvant treatment and at the time of surgery. The present study did not include mutation profiling or characterization of immune infiltrates. We plan to perform these types of analyses in the I-SPY 2 TRIAL, which will potentially have greater power to validate specific associations between molecular data and clinical outcomes.

## Conclusions

Transcriptomic analysis of breast tumor samples before, during and after NAC revealed changes in molecular subtypes as well as genes and pathways associated with response and recurrence. These genes and pathways may serve as candidate biomarkers and therapeutic targets for poor response and may aid in early and real-time modification of the treatment protocol to improve clinical outcomes.

## Additional files

Additional file 1:
**Names of all ethical bodies that approved the study in the various centers involved.**
Additional file 2: Figure S1. CONSORT flowchart of patients and data and Venn diagrams of results for association analyses. (**A**) Gene expression analysis was performed on breast cancer tumors collected before treatment (T1), between 24 and 96 hours after initiation of anthracycline-based neoadjuvant chemotherapy (T2) and at the time of surgery (TS). (**B**) Venn diagram showing overlap of genes associated with chemotherapy response as defined by residual cancer burden (RCB). Breast cancer tumors were collected before treatment (T1) and between 24 and 96 hours after initiation of anthracycline-based neoadjuvant chemotherapy (T2). The change in gene expression between two time points is indicated as T2 − T1. Differential expression analysis between RCB 0/I vs. RCB II/III was performed on expression data from T1, T2 and T2 − T1. (**C**) Venn diagram showing overlap of genes associated with recurrence-free survival (RFS). Gene expression profiling of tumors was performed before treatment (T1) and at the time of surgery (TS). The change in expression of genes between two time points is indicated as TS − T1. Genes associated with RFS was examined using Cox proportional hazards modeling. Additional file 3: Table S1. Differentially expressed genes in matched tumor at pretreatment and at 24 to 96 hours after initiation of chemotherapy (T1 vs. T2). Positive direction indicates upregulation at T2 and vice versa. *FDR* false discovery rate. Additional file 4: Table S2. Ingenuity (**A**) gene ontology enrichment and (**B**) pathway analyses for differentially expressed genes between pretreatment and after first dose of chemotherapy (T1 vs. T2) in matched tumors. Additional file 5: Table S3. Tumor gene expression associated with response at pretreatment (T1) and at 24 to 96 hours after initiation of chemotherapy (T2) and gene expression changes between two time points (T2 − T1). The RCB 0/I column is positive if higher expression level or larger change is observed in the RCB 0/I responder group and negative if higher in the RCB II/III nonresponder group. Additional file 6: Table S4. Ingenuity (**A**) gene ontology enrichment and (**B**) pathway analyses for genes whose expression changed between pretreatment and the first dose of chemotherapy (T2 − T1) and were associated with residual cancer burden. Additional file 7: Table S5. Differentially expressed genes in matched tumors at pretreatment versus after treatment during surgery (T1 vs. TS of non-responders). Positive direction indicates upregulation at TS and vice versa. *FDR* false discovery rate. Additional file 8: Table S6. Ingenuity (**A**) gene ontology enrichment and (**B**) pathway analyses for differentially expressed genes between matched pretreatment and surgical tumors (T1 vs. TS). Additional file 9: Table S7. Tumor gene expression associated with recurrence at pretreatment (T1) and at surgery (TS) and gene expression changes between two time points (TS − T1). Additional file 10: Table S8. Ingenuity (**A**) gene ontology enrichment and (**B**) pathway analyses for genes whose expression changed between pretreatment and surgery (TS − T1) and were significantly associated with recurrence-free survival. Additional file 11: Table S9. Ingenuity (**A**) gene ontology enrichment and (**B**) pathway analyses for genes expressed in residual tumors at surgery (TS) and were significantly associated with recurrence-free survival. Additional file 12: Table S10. Concordance of subtype assignments between two time points. Results from neoadjuvant chemotherapy (NAC) studies in which changes in molecular subtype assignment and response to NAC were evaluated (i.e., pathological complete response (pCR) or no pCR).

## Abbreviations

AC: Anthracycline
ER: Estrogen receptor
H&E: Hematoxylin and eosin
HER2: Human epidermal growth factor receptor 2
HR: Hormone receptor
IGF: Insulin-like growth factor 1
IHC: Immunohistochemical
IL: Interleukin
IPA: Ingenuity Pathway Analysis
I-SPY 1 TRIAL: Investigation of Serial Studies to Predict Your Therapeutic Response with Imaging And moLecular Analysis
LR: Likelihood ratio
NAC: Neoadjuvant chemotherapy
pCR: Pathological complete response
PR: Progesterone receptor
RCB: Residual cancer burden
RFS: Recurrence-free survival
T1: Baseline or pretreatment time point
T2: 24–96 hours after initiation of chemotherapy
TS: Surgery time point
