# Supplementary material for: Serial expression analysis of breast tumors during neoadjuvant chemotherapy reveals changes in cell cycle and immune pathways associated with recurrence and response
Source: Breast Cancer Res. 2015 May 29;17(1):73. doi: 10.1186/s13058-015-0582-3 (PMC4479083; doi:10.1186/s13058-015-0582-3)
Supplement: Additional file 1: — Names of all ethical bodies that approved the study in the various centers involved. [file 13058_2015_582_MOESM1_ESM.docx]

Supplementary Methods

Ethical bodies that approved the study in the various centers involved.

The I-SPY 1 TRIAL was a collaboration of the American College of Radiology Imaging Network (ACRIN), Cancer and Leukemia Group B (CALGB), and the National Cancer Institute (NCI)'s Specialized Programs of Research Excellence (SPORE). The protocol was approved by institutional review boards at all participating institutions as listed below:

1. Georgetown University Hospital Institutional Review Board

2. Memorial Sloan-Kettering Cancer Center Institutional Review Board

3. University of Alabama at Birmingham Institutional Review Board for Human Use

4. University of California San Francisco Committee on Human Research

5. University of Chicago Institutional Review Board

6. University of North Carolina IRB and Office of Human Research Ethics

7. University of Pennsylvania Medical Center IRB Human Research Protections

8. University of Texas, Southwestern Institutional Review Board

9. University of Washington, Seattle Human Subjects Division
