# Supplementary material for: Serial expression analysis of breast tumors during neoadjuvant chemotherapy reveals changes in cell cycle and immune pathways associated with recurrence and response
Source: Breast Cancer Res. 2015 May 29;17(1):73. doi: 10.1186/s13058-015-0582-3 (PMC4479083; doi:10.1186/s13058-015-0582-3)
Supplement: Additional file 2: Figure S1. — CONSORT flowchart of patients and data and Venn diagrams of results for association analyses. (A) Gene expression analysis was performed on breast cancer tumors collected before treatment (T1), between 24 and 96 hours after initiation of anthracycline-based neoadjuvant chemotherapy (T2) and at the time of surgery (TS). (B) Venn diagram showing overlap of genes associated with chemotherapy response as defined by residual cancer burden (RCB). Breast cancer tumors were collected before treatment (T1) and between 24 and 96 hours after initiation of anthracycline-based neoadjuvant chemotherapy (T2). The change in gene expression between two time points is indicated as T2 − T1. Differential expression analysis between RCB 0/I vs. RCB II/III was performed on expression data from T1, T2 and T2 − T1. (C) Venn diagram showing overlap of genes associated with recurrence-free survival (RFS). Gene expression profiling of tumors was performed before treatment (T1) and at the time of surgery (TS). The change in expression of genes between two time points is indicated as TS − T1. Genes associated with RFS was examined using Cox proportional hazards modeling. [file 13058_2015_582_MOESM2_ESM.pptx]

## Slide 1
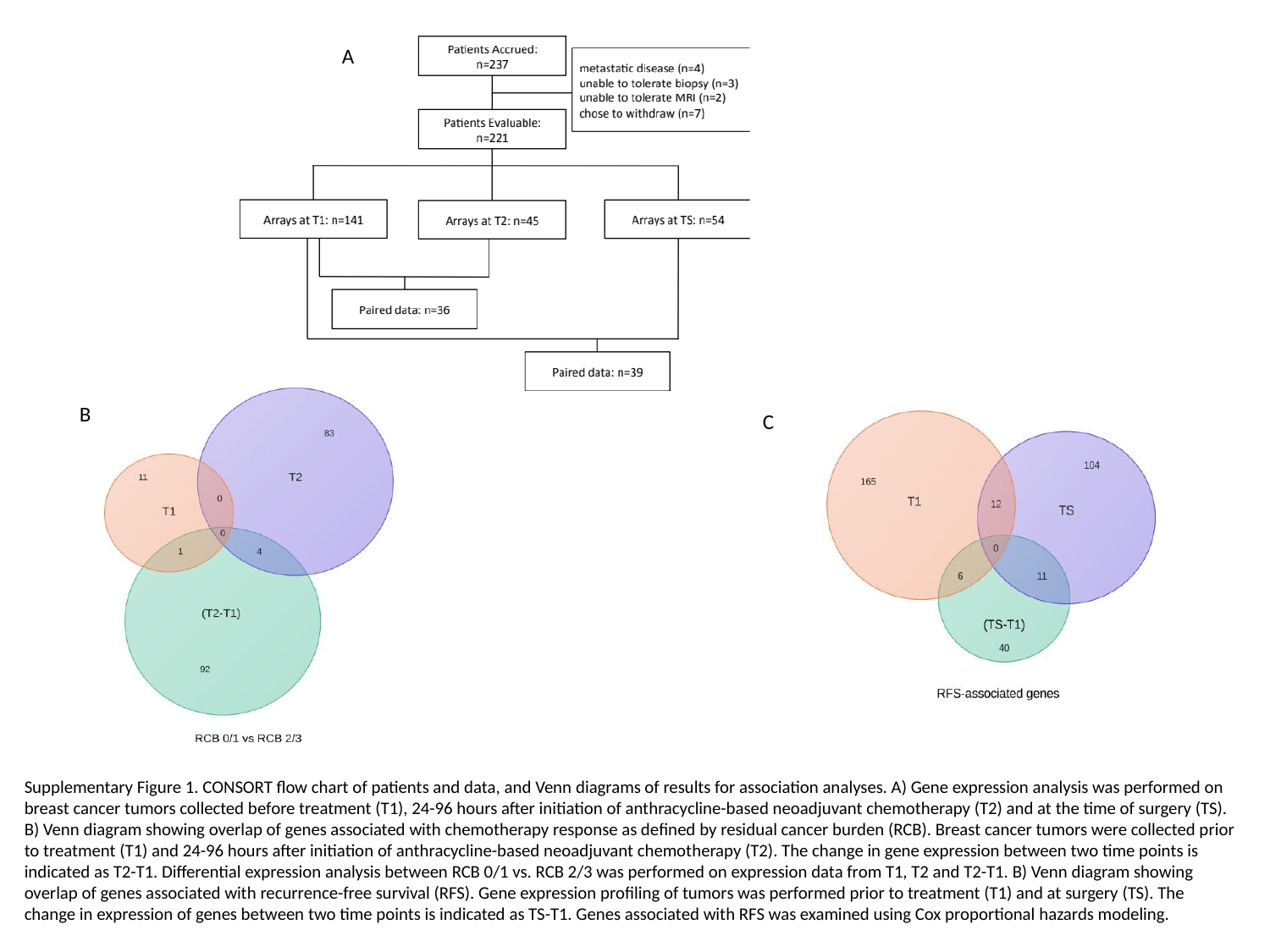

A
B
C
Supplementary Figure 1. CONSORT flow chart of patients and data, and Venn diagrams of results for association analyses. A) Gene expression analysis was performed on breast cancer tumors collected before treatment (T1), 24-96 hours after initiation of anthracycline-based neoadjuvant chemotherapy (T2) and at the time of surgery (TS). B) Venn diagram showing overlap of genes associated with chemotherapy response as defined by residual cancer burden (RCB). Breast cancer tumors were collected prior to treatment (T1) and 24-96 hours after initiation of anthracycline-based neoadjuvant chemotherapy (T2). The change in gene expression between two time points is indicated as T2-T1. Differential expression analysis between RCB 0/1 vs. RCB 2/3 was performed on expression data from T1, T2 and T2-T1. B) Venn diagram showing overlap of genes associated with recurrence-free survival (RFS). Gene expression profiling of tumors was performed prior to treatment (T1) and at surgery (TS). The change in expression of genes between two time points is indicated as TS-T1. Genes associated with RFS was examined using Cox proportional hazards modeling.
