# Supplementary material for: Serial expression analysis of breast tumors during neoadjuvant chemotherapy reveals changes in cell cycle and immune pathways associated with recurrence and response
Source: Breast Cancer Res. 2015 May 29;17(1):73. doi: 10.1186/s13058-015-0582-3 (PMC4479083; doi:10.1186/s13058-015-0582-3)
Supplement: Additional file 3: Table S1. — Differentially expressed genes in matched tumor at pretreatment and at 24 to 96 hours after initiation of chemotherapy (T1 vs. T2). Positive direction indicates upregulation at T2 and vice versa. FDR false discovery rate. [file 13058_2015_582_MOESM3_ESM.docx]

**Supplementary Table 1.** Differentially expressed genes in matched tumor at pretreatment and at 24-96 hours after initiation of chemotherapy (T1vsT2). Positive "Direction" indicates up-regulation at T2 and vice-versa. FDR- false discovery rate.
