# Supplementary material for: Serial expression analysis of breast tumors during neoadjuvant chemotherapy reveals changes in cell cycle and immune pathways associated with recurrence and response
Source: Breast Cancer Res. 2015 May 29;17(1):73. doi: 10.1186/s13058-015-0582-3 (PMC4479083; doi:10.1186/s13058-015-0582-3)
Supplement: Additional file 4: Table S2. — Ingenuity (A) gene ontology enrichment and (B) pathway analyses for differentially expressed genes between pretreatment and after first dose of chemotherapy (T1 vs. T2) in matched tumors. [file 13058_2015_582_MOESM4_ESM.docx]

**Supplementary Table 2.** Ingenuity A) gene ontology enrichment and B) pathway analyses for differentially expressed genes between pretreatment and after first dose of chemotherapy (T1vsT2) in matched tumors.

**A)**

**B)**
