# Supplementary material for: Serial expression analysis of breast tumors during neoadjuvant chemotherapy reveals changes in cell cycle and immune pathways associated with recurrence and response
Source: Breast Cancer Res. 2015 May 29;17(1):73. doi: 10.1186/s13058-015-0582-3 (PMC4479083; doi:10.1186/s13058-015-0582-3)
Supplement: Additional file 5: Table S3. — Tumor gene expression associated with response at pretreatment (T1) and at 24 to 96 hours after initiation of chemotherapy (T2) and gene expression changes between two time points (T2 − T1). The RCB 0/I column is positive if higher expression level or larger change is observed in the RCB 0/I responder group and negative if higher in the RCB II/III nonresponder group. [file 13058_2015_582_MOESM5_ESM.docx]

**Supplementary Table 3.** Tumor gene expression associated with response at pretreatment (T1) and at 24-96 hours after initiation of chemotherapy (T2) and gene expression changes between two time points (T2-T1). The ‘RCB0/1’ column is positive if higher expression level or larger change is observed in the RCB0/1 responder group and negative if higher in the RCB2/3 non-responder group.
