# Supplementary material for: Serial expression analysis of breast tumors during neoadjuvant chemotherapy reveals changes in cell cycle and immune pathways associated with recurrence and response
Source: Breast Cancer Res. 2015 May 29;17(1):73. doi: 10.1186/s13058-015-0582-3 (PMC4479083; doi:10.1186/s13058-015-0582-3)
Supplement: Additional file 7: Table S5. — Differentially expressed genes in matched tumors at pretreatment versus after treatment during surgery (T1 vs. TS of non-responders). Positive direction indicates upregulation at TS and vice versa. FDR false discovery rate. [file 13058_2015_582_MOESM7_ESM.docx]

**Supplementary Table 5.** Differentially expressed genes in matched tumors tumors at pretreatment versus after treatment during surgery (T1vsTS). Positive "Direction" indicates up-regulation at TS and vice-versa. FDR- false discovery rate.
