# Supplementary material for: Serial expression analysis of breast tumors during neoadjuvant chemotherapy reveals changes in cell cycle and immune pathways associated with recurrence and response
Source: Breast Cancer Res. 2015 May 29;17(1):73. doi: 10.1186/s13058-015-0582-3 (PMC4479083; doi:10.1186/s13058-015-0582-3)
Supplement: Additional file 9: Table S7. — Tumor gene expression associated with recurrence at pretreatment (T1) and at surgery (TS) and gene expression changes between two time points (TS − T1). [file 13058_2015_582_MOESM9_ESM.docx]

**Supplementary Table 7.** Tumor gene expression associated with recurrence at pretreatment (T1) and at surgery (TS) and gene expression changes between two time points (TS-T1).
