# Supplementary material for: Serial expression analysis of breast tumors during neoadjuvant chemotherapy reveals changes in cell cycle and immune pathways associated with recurrence and response
Source: Breast Cancer Res. 2015 May 29;17(1):73. doi: 10.1186/s13058-015-0582-3 (PMC4479083; doi:10.1186/s13058-015-0582-3)
Supplement: Additional file 10: Table S8. — Ingenuity (A) gene ontology enrichment and (B) pathway analyses for genes whose expression changed between pretreatment and surgery (TS − T1) and were significantly associated with recurrence-free survival. [file 13058_2015_582_MOESM10_ESM.docx]

**Supplementary Table 8.** Ingenuity A) gene ontology enrichment and B) pathway analyses for genes whose expression changed between pretreatment and surgery (TS-T1) and were significantly associated with recurrence-free survival.

**A)**

**B)**
