# Supplementary material for: Serial expression analysis of breast tumors during neoadjuvant chemotherapy reveals changes in cell cycle and immune pathways associated with recurrence and response
Source: Breast Cancer Res. 2015 May 29;17(1):73. doi: 10.1186/s13058-015-0582-3 (PMC4479083; doi:10.1186/s13058-015-0582-3)
Supplement: Additional file 12: Table S10. — Concordance of subtype assignments between two time points. Results from neoadjuvant chemotherapy (NAC) studies in which changes in molecular subtype assignment and response to NAC were evaluated (i.e., pathological complete response (pCR) or no pCR). [file 13058_2015_582_MOESM12_ESM.docx]

**Supplementary Table 10.** Concordance of subtype assignments between two time points. Results from neoadjuvant chemotherapy (NAC) studies which evaluated changes in molecular subtype assignment and response to NAC, i.e. pathological complete response (pCR) or no PCR.
